# Sex-specific associations between lipids and cognitive decline in the middle-aged and elderly: a cohort study of Chinese adults

**DOI:** 10.1186/s13195-020-00731-1

**Published:** 2020-12-07

**Authors:** Lili Liu, Chen Zhang, Xiaozhen Lv, Xuefeng Lai, Lu Xu, Jingnan Feng, Yongfeng Song, Shengfeng Wang, Siyan Zhan

**Affiliations:** 1 Department of Epidemiology and Biostatistics, School of Public Health, Peking University, 38 Xueyuan Road, Haidian District, Beijing, 100191 China; 2 Department of Endocrinology, Shandong Provincial Hospital Affiliated to Shandong First Medical University, 324 Jing 5 road, Huaiyin District, Jinan, 250021 China; 3 Beijing Dementia Key Laboratory, National Clinical Research Center for Mental Disorders, Peking University Sixth Hospital (Institute of Mental Health), 51 Huayuanbei Road, Haidian District, Beijing, 100191 China; 4 School of Public Health, Peking University, 38 Xueyuan Road, Haidian District, Beijing, 100191 China; 5 Research Center of Clinical Epidemiology, Peking University Third Hospital, 49 Huayuan North Road, Haidian District, Beijing, 100191 China; 6 Center for Intelligent Public Health, Institute for Artificial Intelligence, Peking University, Beijing, 100191 China

**Keywords:** Lipids, Cognitive decline, Sex

## Abstract

**Background:**

Studies regarding the lipid-cognition relationship have increasingly gained popularity but have generated much mixed results. To date, few studies have focused on the difference between sexes.

**Methods:**

This study included 6792 Chinese adults aged over 45 years (women, 48.56%; mean age, 57.28 years), who were free of severe conditions known to affect cognitive function at the baseline (2011). Blood concentrations of total cholesterol (TC), high-density lipoprotein cholesterol (HDL-c), low-density lipoprotein cholesterol (LDL-c), and triglycerides (TG) were assessed at baseline, and both continuous and categorical values were used in final analyses. Global cognitive functions were assessed by the word recall test and the mental status test in 2011, 2013, and 2015, respectively. We graded participants into three groups according to the cognitive change slopes: no decline (≥ 0), moderate decline (median to 0), and severe decline (< median). Sex-specific associations between blood lipids and cognitive decline were analyzed using ordinal logistic models, adjusting for sociodemographic information, lifestyle behaviors, and health status.

**Results:**

Higher baseline TC and LDL-C concentrations exhibited no significant association with 5-year cognitive decline in men but were significantly associated with greater 5-year cognitive decline in women [odds ratio (OR) 1.026, 95% confidence interval (CI) 1.003, 1.050; OR 1.026, CI 1.002, 1.051, respectively]. For higher serum HDL-c levels, a significantly protective effect on cognition was observed in men, but a slightly adverse effect was found in women (not significant after Bonferroni correction). TG presented almost no effect on later cognition in either sex.

**Conclusion:**

Different associations between sexes were observed for the lipid-cognition relationship, and maintaining serum cholesterol levels at an appropriate range may have a positive effect on cognitive health.

## Background

With the population aging rapidly, cognitive impairment has become a serious global public health issue. Approximately 20–30% of Americans and 8% Chinese aged over 65 years are affected by cognitive impairment, ranging from mild deficits to dementia [1, 2]. Given that no reversible therapy is currently available for cognitive impairment, it is crucial to identify potential modifiable risk factors for rapid cognitive decline and thus employ corresponding strategies for early intervention.

Dyslipidemia is a well-recognized risk factor for atherosclerosis as well as cardio-cerebrovascular diseases. Since atherosclerosis and vascular diseases are important contributing factors to cognitive decline and dementia [3, 4], exploring the relationship of dyslipidemia with cognitive decline has aroused increasing interest in recent years. However, the results have always been conflicting. A few epidemiological studies suggested that high serum total cholesterol (TC) levels predicted subsequently greater cognitive decline or the onset of dementia in general populations [5–8], and lipid lowering agents might have protective effects [9]. However, other studies showed no [10–12] or reverse associations [13–15].

Sex differences have been noted in lipid profiles, vascular physiology, specific cognitive domains, and progression of dementia apart from hormone status [16–19], and the prevalence of dementia in women exhibits a 1.65-fold increase compared to that in men in China [20]. Different compositions between sexes might thus explain the contradictory findings of those longitudinal studies related to the lipid-cognition associations. However, such population-based studies that are stratified by sex are limited and have drawn contradictory conclusions [5, 21, 22]. A prior longitudinal study in Sweden, where baseline serum TC, HDL-c, and TG levels were higher in women than men, reported that higher TC in men and lower triglycerides or higher HDL-C in women predicted better maintenance of cognitive abilities [21]. However, another prospective study conducted in China reported no sex difference in the lipid-cognition associations [5]. Conversely, in the Three-City study of French, a hypercholesterolemic pattern in men and a hypolipidemic pattern in women were associated with an increased risk of cognitive decline after a 7-year follow-up [22]. In this study, women also exhibited different baseline serum cholesterol levels compared with men (increased TC, LDL-C, and HDL-c levels, except TG).

In total, gaps in the knowledge of sex-specific associations between lipids and cognition currently exist, especially in the middle-aged population, and the sample sizes of existing cohort studies were relatively small [5, 21, 22]. This study therefore aimed to explore the sex-specific associations of serum lipids with 5-year cognitive decline in a community-based longitudinal study of Chinese elderly individuals derived from the China Health and Retirement Longitudinal Study (CHARLS) based on its large sample, containing middle-aged participants and its thorough and detailed information on the exposure and outcome.

## Methods

### Study population

CHARLS is a nationally representative cohort study of 17,424 individuals recruited at ages over 45 years from 450 communities in China [23]. The baseline survey was conducted in 2011–2012, and a face-to-face computer-assisted personal interviewing was used to collect sociodemographic information, lifestyle behaviors, and health and cognitive status. Among the study participants, 13,978 individuals (78.9%) provided anthropometric and physical performance measures. In this group, blood samples were collected from 11,847 individuals, yielding a response rate of 67%. Two biennial visits were followed in 2013 and 2015 [24], and details of the program were available elsewhere [23].

For this analysis, we excluded CHARLS participants who did not engage in the blood test (*n* = 5861) at baseline and who did not complete cognitive testing at baseline (*n* = 1924). Other exclusion criteria for the original study involved patients suffering from severe diseases or conditions known to affect cognitive function (*n* = 2187) (e.g., depression, malignant tumors, a history of traumatic brain injury, cerebral infarction or cerebrovascular disease, long-term intake of drugs and medication or dietary supplement to improve cognitive function). Consistent with a previous study [5], 660 individuals with a global cognition score of less than or equal to 5 were also excluded due to cognition function being too impaired to be able to complete the cognitive tests or questionnaires (using 5 as the cut-off point to exclude participants with the 5% lowest cognitive scores [5]). The survey was approved by the Institutional Review Board of Peking University, China (IRB00001052-11015). All subjects provided written informed consent to participate at each study visit.

### Lipid measurement

Serum total cholesterol (TC), low-density lipoprotein cholesterol (LDL-C), high-density lipoprotein cholesterol (HDL-C), and triglycerides (TG) were determined at baseline by an automatic analyzer using enzymatic colorimetric test, and all tests were performed at the Youanmen Center for Clinical Laboratory of Capital Medical University [23]. We considered concentrations of TC, LDL-C, HDL-C, and TG regardless of fasting status, given that 92.03% of participants had been fasting > 8 h at the time of blood draw. Blind-duplicate coefficients of variation ranged from 0.7 to 1.8% [25]. We performed a post hoc analysis to see whether higher TC and LDL-C levels were showed in higher HDL-C grades.

### Cognitive assessment

At each study visit, trained study personnel administered the Word Recall Test (WRT) and the Mental Status Test (MST) in a standard order [26, 27]. Similar to the cognitive measurements used in the American Health and Retirement Study (HRS) [23], two composite measures for cognitive functioning were conducted in this study, including the word recall test (WRT) and mental status test (MST): (1) WRT: This test aimed to assess episodic memory by immediate word recall and delayed word recall. Interviewers read a list of ten words only once, and then, the respondents were asked to recall as many of the words as they could in any order (immediate word recall). Then, approximately 4 min later, they were asked to recall the same list words again (delayed word recall). The word recall score is based on the average of the number of correct answers, ranging from 0 to 10. Immediate and delayed recall tests were previously demonstrated to have good construct validity and consistency [28]. (2) MST: This test aimed to assess executive function using the TICS-10 (Telephone Interview for Cognitive Status-10) and figure drawing. The TICS-10 is a well-established and valid measure as the Mini-Mental State Examination (MMSE) used to screen cognitively impaired elderly [29] and involves ten questions, including recalling today’s date (month, day, year), the day of the week and season of the year, and serial 7 subtraction from 100 (up to five times). This dimension score was calculated based on the number of correct answers, ranging from 0 to 10. In the figure drawing, the participants were asked to replicate a figure as similarly as possible, and interviewers would score the answer as 1 if the participants successfully completed this task. Those who failed to complete this task received a score of 0. The MST was performed once for each participant at each visit.

We used the sum of both of the scores of WRT (0 to 10) and MST (0 to 11) as the global cognition score to represent the respondent’s comprehensive cognitive status, with scores ranging from 0 to 21 and a higher score indicating better cognitive function [23]. Consistent with previous studies [22, 30], slopes of cognitive decline were first calculated by linear regressions directly to quantify the changes of cognition during 2011–2015 (not adjusting for age and education). To promote the clinical interpretability of outcome, three groups of participants were then graded according to the cognitive change slopes: no decline (≥ 0), moderate decline (median to 0), and severe decline (< median).

### Covariate assessment

Similar to previous studies [5, 6, 31], potential confounders included age (years), education (illiterate/primary school/middle school and above), marriage (married/divorced/single), residence (urban/rural), leisure time social activity (active/inactive/none), health insurance status (yes/no, as a proxy for socioeconomic status and access to health care), alcohol use (current/former/never), smoking status (current/former/never), hypertension (yes/no), diabetes (yes/no), and lipid-lowering medication use (yes/no). Body mass index (BMI) was calculated as body weight (kg) divided by the square of height (m^2^). Diabetes was defined as a self-reported physician diagnosis of diabetes, ≥ 126 mg/dL fasting glucose, ≥ 200 mg/dL non-fasting glucose, or use of diabetes medications [32]. Hypertension was determined as self-reported physician diagnosis of hypertension, measured systolic blood pressure ≥ 140 mmHg, measured diastolic blood pressure ≥ 90 mmHg, or use of antihypertensive medications. Additional variables used in sensitivity analyses included fasting status at baseline blood draw (> 8 h, yes/no). After the selection of covariates, we finally adjusted all these potential confounders in the main analyses. However, in the subgroup analyses of age or education, we did not further correct the age or education.

### Statistical analyses

The Student *t* test and the chi-squared test for categorical variables were used to identify basic differences between sexes. Separate ordinal logistic regression models were used to consider whether baseline lipid levels were associated with later cognitive decline in males and females. Lipid profiles were first put in models as continuous variables per 10 mg/dl. Categorical lipid variables, defined by the *Third Report of the Expert Panel on Detection, Evaluation, and Treatment of High Cholesterol in Adults (ATP-III)* [33], were analyzed in the same models to promote the clinical interpretability. According to a prior study [34], a minor elevation of cholesterol levels, even in a normal range, was associated with later endothelial dysfunction, which thus might increase the risk of vascular disease caused by elevated cholesterol. Similarly, the fluctuation of lipids within the normal range might also influence the later cognition, and thus, we finally reported both continuous and categorical results due to the clinical significance and the comprehensiveness of results [31]. Furthermore, given that the cognitive decline rate of the elderly group was more rapid than that of middle-aged people [5, 35], we also conducted subgroup analyses to determine whether there was support for effect measure modification of age. Consistent with previous studies [36, 37], we chose a cut-off of 60 years for these subgroup analyses. To make our results nationally representative, a sample weight, which was calculated using an inverse probability method, was used under the correction for household and individual non-response as well as non-participation in the blood collection [23]. All the multivariable analyses in our study were weighted.

For our primary analyses, we used a complete case approach to address missing data. In the sensitivity analysis, we examined associations after (1) imputing those with a global cognition score of less than or equal to 5 only; (2) additionally imputing those with severe diseases or conditions known to affect cognitive function; (3) not adjusting for obesity, diabetes, and hypertension; (4) excluding participants with obesity, diabetes, or hypertension; and (5) not adjusting for marital and health insurance status. Given that fasting status can impact lipid values, we repeated our primary analyses after restricting them to individuals who were fasting > 8 h before blood draw.

Stata 15.0 (StataCorp LP) was used for all analyses, and formal hypothesis testing was 2-sided with a significant level of 0.05. Throughout the analysis, we report 95% confidence intervals and a *p* value of < 0.05 was considered to be statistically significant. We used the Bonferroni test for multiple corrections.

## Results

### Subjects

A total of 6792 individuals were included in the analysis, including 3494 men and 3298 women, with a mean (SD) age of 59.26 (8.95) years for men and 57.28 (8.71) years for women (*p* < 0.001). At baseline, women were more frequently divorced or single and with more living in town (*p* < 0.001). The overall mean concentrations of TC, LDL-C, HDL-C, and TG were 192.57 (38.19) mg/dl, 116.57 (34.58) mg/dl, 50.69 (14.71) mg/dl, and 128.79 (83.87) mg/dl, respectively, and all indices were significantly higher in women except HDL-C. According to the *ATP-III*, the abnormal rate of these four indices were 38.87%, 11.88%, 24.60%, and 26.30%, respectively. Clearly, the abnormal rates of the four lipids indices in women were significantly increased compared with those in men (Table 1). Moreover, higher TC and LDL-C levels were noted in the high HDL-C grade (Supplementary Table 1).

**Table 1:** Descriptive statistics for eligible individuals at baseline

Characteristics	Overall, *N* = 6792	Male, *n* = 3494	Female, *n* = 3298	*P* value
Age, mean (SD)	58.30 (8.89)	59.26 (8.95)	57.28 (8.71)	< 0.001
In married, *n* (%)	6137 (90.36)	3246 (92.90)	2891 (87.66)	< 0.001
Urban, *n* (%)	2740 (40.34)	1327 (37.98)	1413 (42.84)	< 0.001
Education, *n* (%)				
Illiterate	1364 (20.08)	313 (8.96)	1051 (31.87)	< 0.001
Primary school	2887 (42.51)	1608 (46.02)	1279 (38.78)	
Middle school and above	2541 (37.41)	1573 (45.02)	968 (29.35)	
Smoking, *n* (%)				
Never	3915 (57.65)	874 (25.01)	3041 (92.24)	< 0.001
Former	1175 (17.30)	1035 (29.63)	140 (4.24)	
Current	1701 (25.05)	1585 (45.36)	116 (3.52)	
Alcohol use, *n* (%)				
Never	4026 (59.28)	1194 (34.17)	2832 (85.87)	< 0.001
Former	399 (5.87)	329 (9.42)	70 (2.12)	
Current	2367 (34.85)	1971 (56.41)	396 (12.01)	
Lipid-lowering medication using, *n* (%)	161 (1.71)	57 (1.69)	59 (1.73)	0.900
Health insurance, *n* (%) ^a^	6438 (94.96)	3329 (95.39)	3109 (94.50)	0.095
Socially active, *n* (%)				
None	3925 (57.79)	2049 (58.64)	1876 (56.88)	0.314
Inactive	1893 (27.87)	949 (27.16)	944 (28.63)	
Active	974 (14.34)	496 (14.20)	478 (14.49)	
Body mass index (kg/m^2^), *n* (%) ^b^				
< 18.5	345 (5.21)	194 (5.71)	151 (4.69)	< 0.001
18.5~23.9	3378 (51.06)	1950 (57.37)	1428 (44.39)	
24.0~27.9	2080 (31.44)	952 (28.01)	1128 (35.06)	
> 28.0	813 (12.29)	303 (8.91)	510 (15.86)	
Diabetes, *n* (%)	931 (13.72)	478 (13.69)	453 (13.75)	0.947
Hypertension, *n* (%)	2717 (40.00)	1355 (38.78)	1362 (41.30)	0.034
Total cholesterol (mg/dl), mean (SD)	192.57 (38.19)	188.25 (38.31)	197.17 (37.52)	< 0.001
< 200, *n* (%)	4081 (61.13)	2258 (65.62)	1823 (56.35)	< 0.001
200~239, *n* (%)	1891 (28.33)	882 (25.63)	1009 (31.19)	
240+, *n* (%)	704 (10.54)	301 (8.75)	403 (12.46)	
LDL-c (mg/dl), mean (SD)	116.57 (34.58)	113.04 (34.38)	120.31 (34.39)	< 0.001
< 100, *n* (%)	2152 (32.26)	1242 (36.14)	910 (28.15)	< 0.001
100~129, *n* (%)	2392 (35.86)	1221 (35.53)	1171 (36.22)	
130~159, *n* (%)	1448 (21.72)	695 (20.22)	753 (23.29)	
160~189, *n* (%)	501 (7.51)	195 (5.67)	306 (9.46)	
190+, *n* (%)	177 (2.65)	84 (2.44)	93 (2.88)	
HDL-c (mg/dl), mean (SD)	50.69 (14.71)	50.35 (15.56)	51.04 (13.73)	0.054
< 40, *n* (%)	1643 (24.60)	948 (27.54)	695 (21.48)	< 0.001
40~59, *n* (%)	3505 (52.48)	1713 (49.75)	1792 (55.38)	
60+, *n* (%)	1531 (22.92)	782 (22.71)	749 (23.14)	
Triglycerides (mg/dl), mean (SD)	128.79 (83.87)	124.33 (83.88)	133.52 (83.61)	< 0.001
< 150, *n* (%)	4908 (73.70)	2605 (75.93)	2303 (71.34)	< 0.001
150~199, *n* (%)	842 (12.65)	382 (11.13)	460 (14.25)	
200+, *n* (%)	909 (13.65)	444 (12.94)	465 (14.41)	

*LDL-c* low-density lipoprotein cholesterol, *HDL-c* high-density lipoprotein cholesterol

^a^12 missing

^b^176 missing

### Cognitive decline

The medians of cognitive change slopes used to divide participants were − 0.75. − 0.75 and − 0.38 for the three cognitive scores, respectively. At baseline, men had higher scores of global cognition [12.31 (3.07) versus 11.34 (3.38), *P* < 0.001] and mental status [8.51 (2.30) versus 7.45(2.62), *P* < 0.001] compared with women. However, for episodic memory, women scored better than men [3.89 (1.63) versus 3.80 (1.57), *P* = 0.018]. In view of the cognitive decline during 2011–2015, no significant differences were noted between men and women among groups of no decline, moderate decline and severe decline. Furthermore, the proportion of decline (moderate and severe) on three cognitive indices in men was comparable to that in women (Table 2).

**Table 2:** Cognitive descriptions of eligible individuals

	Overall, *N* = 6792	Male, *n* = 3494	Female, *n* = 3298	*P* value
Cognition at baseline, mean (SD)				
Global cognition	11.84 (3.26)	12.31 (3.07)	11.34 (3.38)	< 0.001
Mental status	7.99 (2.52)	8.51 (2.30)	7.45 (2.62)	< 0.001
Episodic memory	3.85 (1.60)	3.80 (1.57)	3.89 (1.63)	0.018
Cognition at 2015, mean (SD)				
Global cognition	10.96 (3.93)	11.51 (3.60)	10.38 (4.16)	< 0.001
Mental status	7.39 (2.91)	7.96 (2.65)	6.79 (3.04)	< 0.001
Episodic memory	3.47 (1.79)	3.44 (1.74)	3.50 (1.83)	0.226
Absolute cognitive decline during 2011–2015, mean (SD)				
Global cognition	0.88 (3.44)	0.88 (3.38)	0.88 (3.50)	0.969
Mental status	0.58 (2.71)	0.59 (2.67)	0.57 (2.76)	0.781
Episodic memory	0.37 (1.86)	0.37 (1.83)	0.37 (1.89)	0.982
Groups of global cognition change, *n* (%) ^a^				
No decline	3119 (47.55)	1612 (47.87)	1507 (47.20)	0.859
Moderate decline	1791 (27.30)	914 (27.15)	877 (27.46)	
Severe decline	1650 (25.15)	841 (24.98)	809 (25.34)	
Groups of mental status change, *n* (%) ^b^				
No decline	3637 (54.67)	1898 (55.37)	1739 (53.93)	0.499
Moderate decline	1531 (23.02)	779 (22.72)	752 (23.33)	
Severe decline	1484 (22.31)	751 (21.91)	733 (22.74)	
Groups of episodic memory change, *n* (%) ^c^				
No decline	3442 (51.74)	1769 (51.60)	1673 (51.89)	0.884
Moderate decline	1683 (25.30)	876 (25.55)	807 (25.03)	
Severe decline	1527 (22.96)	783 (22.85)	744 (23.08)	

^a^232 missing, no decline (slope of change ≥ 0), moderate decline (slope of change: median to 0) and severe decline (slope of change < median)

^b^140 missing, no decline (slope of change ≥ 0), moderate decline (slope of change: median to 0) and severe decline (slope of change < median)

^c^140 missing, no decline (slope of change ≥ 0), moderate decline (slope of change: median to 0) and severe decline (slope of change < median)

### Lipids and cognitive decline

Overall, after multivariate adjustment, higher baseline TC or LDL-C level was significantly associated with a greater 5-year decline in global cognition [OR 1.026, 95% CI 1.003, 1.050; OR 1.026, 95% CI 1.002, 1.051] in women, whereas no significant association was observed in men. Furthermore, significant associations were also found between higher baseline TC or LDL-C level and faster 5-year decline on the mental status [OR 1.030, 95% CI 1.007, 1.054; OR 1.034, 95% CI 1.010, 1.059] in women, and similarly, no significant association was found in men. In general, no significant association was observed between HDL-C and TG and 5-year cognitive decline. However, we noted that higher HDL-C exhibited a marginally significant association with slower 5-year decline in mental status in men (OR 0.948, 95%CI 0.900, 1.000, *P* = 0.051) and demonstrated an adverse effect in women (OR 1.044, 95%CI 0.981, 1.110, *P* = 0.173). No significant association was found overall between lipids and episodic memory both in men and women (Table 3).

**Table 3:** Adjusted odds ratio in 5-year cognitive decline per 10 mg/dL lipids at baseline, OR (95%CI)

	Global cognition^†^		Mental status^†^		Episodic memory^†^		
	Male	Female	Male	Female	Male	Female	
Total cholesterol	1.000 (0.979, 1.021)	1.026 (1.003, 1.050) *	0.997 (0.976, 1.018)	1.030 (1.007, 1.054) **	1.000 (0.978, 1.022)	1.003 (0.981, 1.026)	
LDL-c	0.995 (0.972, 1.018)	1.026 (1.002, 1.051) *	0.994 (0.971, 1.017)	1.034 (1.010, 1.059) **	1.001 (0.978, 1.025)	0.997 (0.973, 1.022)	
HDL-c	0.956 (0.907, 1.007)	1.059 (0.995, 1.128)	0.948 (0.900, 1.000)	1.044 (0.981, 1.110)	0.970 (0.921, 1.022)	1.059 (0.997, 1.125)	
Triglycerides	1.005 (0.995, 1.016)	0.998 (0.989, 1.007)	1.004 (0.994, 1.015)	0.999 (0.990, 1.009)	1.001 (0.991, 1.011)	0.999 (0.988, 1.011)	

*OR* odds ratio, *CI* confidence interval, *LDL-c* low-density lipoprotein cholesterol, *HDL-c* high-density lipoprotein cholesterol

*P < 0.05

**P < 0.01

^†^Adjusted for baseline age, education, marital status, registered residence, body mass index, alcohol use, smoking status, diabetes, hypertension, social activity, health insurance status, and lipid-lowering medication use

Before Bonferroni correction, analyses compared with lipid categories indicated a significant association between elevated TC and greater 5-year decline of mental status in women. The elevation of LDL-C was associated with greater 5-year decline on the global cognition and the mental status in women, both showing a linear-dose response pattern. No significant association was found between lipid categories of baseline TC and LDL-C levels and the subsequent 5-year cognitive decline in men. Elevated HDL-C was associated with slower 5-year cognitive decline in men on global cognition and the mental status but it showed an adverse effect on global cognition in women. However, after Bonferroni correction, only the association between LDL-C and mental status remained. In total, TG did not exhibit a significant association with any of the three cognitive indices regardless of Bonferroni correction (Table 4).

**Table 4:** Adjusted odds ratio in 5-year cognitive change across ATP-III lipid categories at baseline, OR (95%CI)

	Global cognition^†^			Mental status^†^			Episodic memory^†^	
	Male	Female		Male	Female		Male	Female
Total cholesterol (mg/dl)								
< 200	ref	ref		ref	ref		ref	ref
200~239	1.011 (0.848, 1.206)	1.010 (0.833, 1.226)		1.179 (0.983, 1.414)	1.175 (0.972, 1.421)		1.035 (0.869, 1.233)	0.914 (0.752, 1.111)
240+	1.051 (0.807, 1.369)	1.242 (0.965, 1.600)		0.889 (0.681, 1.162)	1.295 (1.002, 1.675) ^b^		1.040 (0.780, 1.387)	1.014 (0.791, 1.300)
LDL-c (mg/dl)								
< 100	ref	ref		ref	Ref		ref	ref
100~129	0.979 (0.813, 1.178)	1.308 (1.051, 1.627) ^b^		0.996 (0.827, 1.199)	1.302 (1.047, 1.618) ^b^		1.004 (0.835, 1.207)	1.207 (0.973, 1.496)
130~159	0.918 (0.727, 1.160)	1.291 (1.017, 1.639) ^b^		0.984 (0.776, 1.248)	1.357 (1.071, 1.720) ^a^		0.943 (0.748, 1.189)	1.058 (0.828, 1.351)
160~189	0.976 (0.696, 1.367)	1.377 (1.002, 1.891) ^b^		0.873 (0.611, 1.249)	1.491 (1.076, 2.066) ^b^		1.134 (0.790, 1.629)	1.120 (0.819, 1.531)
190+	0.998 (0.644, 1.546)	1.424 (0.933, 2.172)		0.807 (0.505, 1.289)	1.283 (0.838, 1.967)		1.283 (0.802, 2.054)	0.903 (0.576, 1.415)
HDL-c (mg/dl)								
< 40	ref	ref		ref	ref		ref	ref
40~59	0.822 (0.679, 0.994) a	1.159 (0.928, 1.447)		0.808 (0.665, 0.980) ^b^	1.161 (0.933, 1.443)		0.861 (0.711, 1.042)	1.087 (0.873, 1.354)
60+	0.794 (0.629, 1.001)	1.294 (1.002, 1.671) ^b^		0.769 (0.614, 0.964) ^b^	1.246 (0.967, 1.606)		0.898 (0.711, 1.134)	1.245 (0.966, 1.605)
Triglycerides (mg/dl)								
< 150	ref	ref		ref	ref		ref	ref
150~199	0.981 (0.766, 1.257)	1.015 (0.795, 1.296)		1.008 (0.781, 1.302)	1.184 (0.938, 1.494)		0.870 (0.675, 1.120)	0.800 (0.630, 1.015)
200+	1.088 (0.863, 1.371)	0.836 (0.663, 1.054)		1.097 (0.864, 1.394)	0.955 (0.760, 1.200)		1.043 (0.819, 1.329)	0.804 (0.628, 1.031)

*OR* odds ratio, *CI* confidence interval, *LDL-c* low-density lipoprotein cholesterol, *HDL-c* high-density lipoprotein cholesterol

^†^Adjusted for baseline age, education, marital status, registered residence, body mass index, alcohol use, smoking status, diabetes, hypertension, social activity, health insurance status, and lipid-lowering medication use

^a^*P* < 0.05 after the Bonferroni correction

^b^*P* < 0.05 before the Bonferroni correction

### Sensitivity and subgroup analyses

Results of our sensitivity analyses were substantially consistent with primary findings, including restricting participants to those with fasting > 8 h; imputing those with a low global cognition score or those with severe conditions; not adjusting for obesity, diabetes, hypertension, marital status and health insurance; and excluding participants with obesity, diabetes, or hypertension (Table 5, Supplementary Table 3).

**Table 5:** Adjusted odds ratio in 5-year cognitive change per 10 mg/dL lipids at baseline across all sensitivity analyses, OR (95%CI)

	Global cognition^†^			Mental status^†^			Episodic memory^†^		
	Male	Female		Male	Female		Male	Female	
**Sensitivity: Restrict to those fasting > 8 h**									
Total cholesterol	1.00 (0.98, 1.02)	1.02 (1.00, 1.05)		1.00 (0.98,1.02)	1.03 (1.00, 1.05) *		1.00 (0.98, 1.02)	1.00 (0.98, 1.02)	
LDL-c	1.00 (0.98, 1.02)	1.03 (1.00, 1.05) *		1.00 (0.98, 1.02)	1.04 (1.01, 1.06) **		1.00 (0.98, 1.03)	1.00 (0.97, 1.02)	
HDL-c	0.95 (0.90, 1.00)	1.07 (1.00, 1.14)		0.94 (0.89,0.99) *	1.04 (0.98, 1.11)		0.97 (0.92, 1.02)	1.06 (0.99, 1.13)	
Triglycerides	1.01 (0.99, 1.02)	0.99 (0.98, 1.00)		1.00 (0.99, 1.02)	1.00 (0.99, 1.01)		1.00 (0.99, 1.01)	1.00 (0.99, 1.01)	
**Sensitivity: Impute those with a global cognition score of less than or equal to 5**									
Total cholesterol	1.00 (0.98, 1.02)	1.02 (1.00, 1.04) *		1.00 (0.98, 1.02)	1.03 (1.01, 1.05) *		1.00 (0.98, 1.02)	1.00 (0.98, 1.03)	
LDL-c	1.00 (0.97, 1.02)	1.02 (1.00, 1.04)		0.99 (0.97, 1.02)	1.03 (1.01, 1.06) **		1.00 (0.98, 1.03)	1.00 (0.97, 1.02)	
HDL-c	0.96 (0.91, 1.01)	1.04 (0.98, 1.10)		0.95 (0.90, 1.00) *	1.04 (0.98, 1.11)		0.97 (0.92, 1.02)	1.06 (1.00, 1.13)	
Triglycerides	1.00 (0.99, 1.01)	1.00 (0.99, 1.01)		1.00 (0.99, 1.01)	1.00 (0.99, 1.01)		1.00 (0.99, 1.01)	1.00 (0.99, 1.01)	
**Sensitivity: Impute those with severe diseases or conditions known to affect cognitive function**									
Total cholesterol	1.00 (0.98, 1.02)	1.02 (1.00, 1.03)		1.00 (0.98, 1.02)	1.03 (1.01, 1.05) *		1.00 (0.98, 1.02)	1.00 (0.98, 1.03)	
LDL-c	1.00 (0.98, 1.02)	1.01 (0.99, 1.03)		0.99 (0.97, 1.02)	1.03 (1.01, 1.06) **		1.00 (0.98, 1.03)	1.00 (0.97, 1.02)	
HDL-c	0.96 (0.91, 1.00)	1.02 (0.97, 1.07)		0.95 (0.90, 1.00) *	1.04, 0.98, 1.11)		0.97 (0.92, 1.02)	1.06 (1.00, 1.13)	
Triglycerides	1.00 (0.99, 1.01)	1.00 (0.99, 1.01)		1.00 (0.99, 1.01)	1.00 (0.99, 1.01)		1.00 (0.99, 1.01)	1.00 (0.99, 1.01)	

*OR* odds ratio, *CI* confidence interval, *LDL-c* low-density lipoprotein cholesterol, *HDL-c* high-density lipoprotein cholesterol

*P < 0.05

**P < 0.01

^†^Adjusted for baseline age, education, marital status, registered residence, body mass index, alcohol use, smoking status, diabetes, hypertension, social activity, health insurance status, and lipid-lowering medication use

**Fig. 1:**
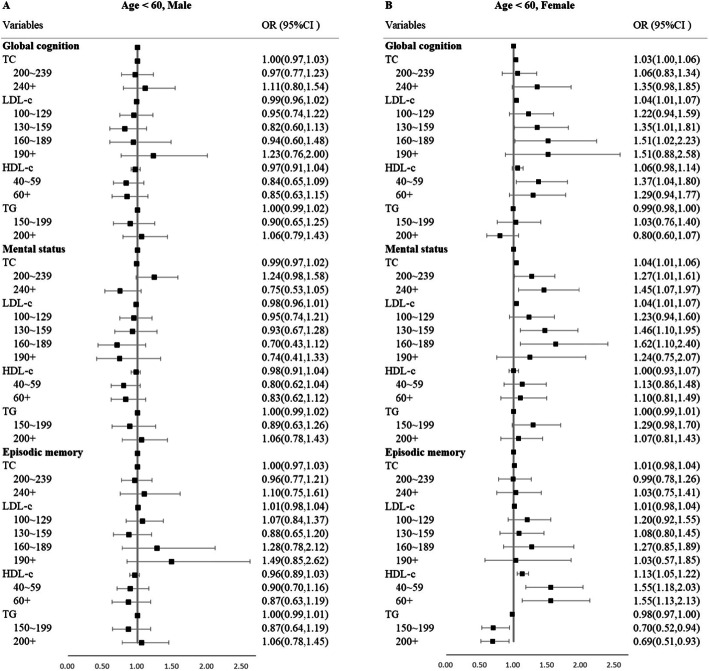
Forest plot of subgroup analyses for age < 60, male (**a**), and age < 60, female (**b**), in ordinal logistic regressions. OR, odds ratio; CI, confidence interval. Adjusting for baseline education, marital status, registered residence, body mass index, alcohol use, smoking status, diabetes, hypertension, social activity, health insurance status, and lipid-lowering medication use

**Fig. 2:**
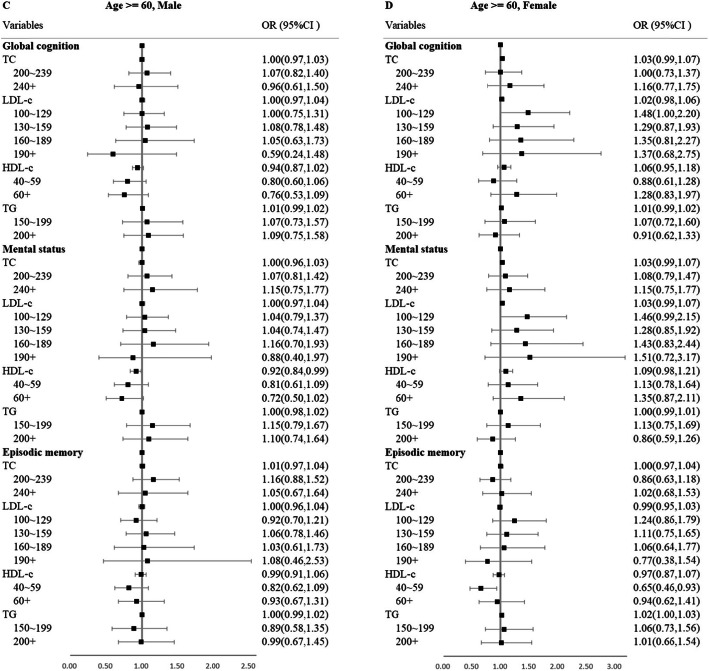
Forest plot of subgroup analyses for age ≥ 60, male (**c**), and age ≥ 60, female (**d**), in ordinal logistic regressions. OR, odds ratio; CI, confidence interval. Adjusting for baseline education, marital status, registered residence, body mass index, alcohol use, smoking status, diabetes, hypertension, social activity, health insurance status, and lipid-lowering medication use

In the subgroup analysis of age, after Bonferroni correction, elevated TC was significantly associated with greater 5-year decline of global cognition among female individuals < 60 years. Elevated LDL-C was significantly associated with greater 5-year decline of global cognition and mental status, and higher HDL-C was associated with faster 5-year decline of episodic memory (Fig. 1). However, no other significant associations were found among male individuals < 60 years and all participants ≥ 60 years (Fig. 2).

## Discussion

In this study, different impacts of the baseline lipid profile on later cognitive decline were observed between sexes over a 5-year period. We found that higher serum TC and LDL-C levels were significantly associated with greater cognitive decline in global cognition and mental status in women. However, these associations were not found in men. Furthermore, a significant protective effect was also observed for higher serum HDL-C levels on cognition in men, but a slightly adverse effect was found in women. TG exhibited almost no effect on cognition in both sexes.

In our study, higher TC and LDL-C levels were significantly associated with greater 5-year cognitive decline in women. This finding was levels consistent with that of a longitudinal study focused on 1037 postmenopausal women [38], and similar results were also shown in another cohort study that included 1159 Chinese participants over 60 years of age [5]. Actually, there are two potential explanations for this finding: (1) the atherosclerosis caused by higher TC and LDL-c levels, subsequently induced cerebral hypoperfusion, which played a significant role in the acceleration of cognitive decline [39, 40], and (2) higher TC levels might modulate enzymatic processing of the amyloid precursor protein and accelerate the deposition of β-amyloid in brain, thus increasing the cognitive decline [41, 42]. However, different findings were noted compared to our study. The WHAS II study that followed 436 community-dwelling women aged 70–79 years for 9 years indicated that no relationship existed between baseline lipids and cognitive decline [43]. However, the participants in this study who had survived to old age would suffer from more complications and were more selective; thus, these women were not susceptible to the adverse effects of higher lipid levels [43]. This result was consistent with that in our subgroup analyses of age, namely, no significant lipid-cognition association was found in individuals ≥ 60 years. Furthermore, it should be noted that its sample size was relatively small; thus, the statistical power might not be sufficient to detect a moderate effect [44]. A similar phenomenon also occurred, when using TC as a categorical variable instead of a continuous variable to explore TC-cognition associations in women [44].

In fact, these effects of TC and LDL-C were exclusively found in women rather than in men, revealing a distinct sex difference for the effect of cholesterol, which also existed in previous studies [21, 22]. Three possible mechanisms were considered. First, the vascular physiology naturally differs between sexes. Compared to men, women have smaller arterial sizes and increased vascular remodeling under atherosclerosis [19], which may cause more microvascular damages and hypoperfusion in the brain [45]. White matter lesions occur more commonly in women compared with men, which may further support this point of view [46]. Second, different hormone levels changes between sexes may be another possible reason. Gonadal hormones (e.g., estradiol and testosterone) serve neuroprotective effects across the human lifespan [47]. Generally, women will experience a dramatic loss of estradiol following menopause, whereas testosterone gradually decline in men as age increases [48]. Thus, given the attenuated neuroprotective effect of estrogen, women seemed to be more vulnerable to the adverse effects of high lipid levels on cognition. Third, as the cerebral brain volume is generally greater in men than in women (~ 10%) [49], the concept of brain reserve was proposed by Katzman et al. [50], suggesting that subjects with larger brain reserve have greater capacity to withstand more pathological conditions. Therefore, compared to women, men were expected to have stronger abilities to fight against the adverse pathological progression of hyperlipidemia. However, an opposite viewpoint was also proposed by Sundermann et al. that women would have a greater cognitive reserve than men due to their innate advantages of verbal memory [51]. However, given that a precise mechanism has not been reported to date, more studies are needed to explore the potential mechanisms for this sex difference in lipid-cognition associations.

Regarding serum HDL-C, no significant effect on cognitive decline was found in men or women after Bonferroni correction, which is consistent with a few prior studies [5, 6, 8]. However, of note, before correction, a protective effect of HDL-C on global cognition and mental status was observed in men, which is consistent with the results of Japanese cohort study [52]. HDL-C can transport excess cholesterol in peripheral tissue to the liver and thus prevent atherosclerosis, which may contribute to maintaining cognition. However, a slightly adverse effect of HDL-C on global cognition in women was also observed, which is consistent with a Chinese cohort study [31]. This novel finding of HDL-C (showing different effects between sexes) was not previously reported. After a systematic literature review, we reasonably hypothesized that this discrepancy might be caused by the different effects of the composition of HDL particles on cognition. HDL particles, a mass of substances in heterogeneous sizes and structures, have various biological functions [53]. Among these particles, small and dense particles were considered to have better atheroprotective properties [53] and reduce the total risk of stroke [54]. In general, men have more small HDL particles compared with women [55, 56], thereby yielding a protective impact of HDL-C on cognition in men. However, in women, these particles were associated with a harmful effect in this study, especially in the HDL-C ≥ 60 mg/dl group. As shown in our study, higher TC and LDL-C levels were noted in these subjects, the adverse effect of which might outweigh the weak protective effect of small HDL particles. Women were more likely to have higher TC and LDL-C levels than men with similar HDL-c levels [57], which would further lead to the contrary impact of HDL-C between sexes in this study.

Of note, all the significant associations mentioned above were merely observed for global cognition and mental status. Even though the global cognition outcomes of this study might be driven by mental status outcomes, but separately, there was indeed no effect found in the episodic memory, suggesting that mental status might be more likely to be affected by lipids. In CHARLS, the mental status test provided a measure of attention, numerical ability and time orientation, which was dominated by the frontal areas in the brain [58]. Based on this notion, there was an underlying possibility that changes in lipids were more likely to affect the frontal areas, while this hypothesis should be further tested [22]. However, our results of mental status were in line with those of the Three-City study, which indicated that frontal executive might be more vulnerable in early cognitive impairment [22]. But it should be noted that we also found very few positive signals on episodic memory in our later subgroup analyses.

According to the subgroup analyses of age, the significant influences of lipids on cognition were mainly noted in younger people (< 60 years), whereas the effects seemed to disappear in the elderly group (≥ 60 years). Consistent with our finding, a recent meta-analysis including 17 cohort studies with 23,338 participants demonstrated that higher serum cholesterol was associated with greater cognitive decline or any dementia in midlife (40–60 years), but no association was found in late-life (> 60 years) [59]. As individuals aged, the effect of TC on cardiovascular diseases is gradually attenuated [60, 61]. Aging might also lead to several changes in the arterial wall that would later decrease its susceptibility to cholesterol levels in the blood [61]. Additionally, due to the deterioration in brain structure and function, cognitive decline in the elderly mainly occurred naturally with aging, which might take place earlier than the effect of lipids on cognition. Moreover, it also should be noted that the proportion of illiterate females in CHARLS was higher than that of men, which is consistent with the 2010 educational status of China in the middle-aged and elderly [62]. As high education levels have been proposed as a proxy for cognitive reserve and are an important protective factor for cognition [63, 64], this large educational difference might have significant impacts on later cognition between sexes. However, lipid-cognition associations in this study remained after correction for education by multivariate adjustment, indicating the potential effect of lipids on later cognition. Furthermore, subgroup analyses based on education showed that the associations between lipids and cognition varied among different educational groups and mainly occurred in females with high educational level, indicating that specific lipid management is needed for both sexes based on educational background [31].

### Limitations

A few limitations existed in this study. First, the cognitive domains tested in this study were relatively limited. However, to the best of our knowledge, mental status and episodic memory can be used to represent the majority domains of cognitive functions [65]. Second, we could not exclude the possibility of residual confounders due to the sex differences on baseline education and other unknown vascular risk factors as well as the absence of information on APOE genotype and dietary cholesterol intake. However, after the multivariable adjustment, the majority of potential confounding effect would be controlled. Third, the lack of imaging diagnoses and molecular markers for neurodegenerative diseases limited our explorations of the mechanism linking high lipid levels and cognitive decline.

## Conclusion

In conclusion, our study suggested that women tended to be more susceptible to adverse effects of higher TC and LDL-C levels on global cognition and mental status. Higher serum HDL-C levels might have beneficial effects on cognition in men but adverse effects in women. Further work is needed to explore the potential mechanisms of the sex-specific associations between lipids and cognitive decline. The results from our study suggested that lipids are a potential treatable factor and maintaining serum cholesterol levels at an appropriate range may have a positive effect on cognitive health.

## Supplementary information

**Additional file 1: Supplementary Table 1.** TC, LDL-c levels in different HDL-c grades. **Supplementary Table 2.** Adjusted odds ratio in 5-year cognitive change in different plasma lipid levels across education levels, OR (95%CI. Abbreviations: OR, odds ratio; CI, confidence interval; LDL-c, low-density lipoprotein cholesterol; HDL-c, high-density lipoprotein cholesterol. ^†^ Adjusted for baseline age, marital status, registered residence, body mass index, alcohol use, smoking status, diabetes, hypertension, social activity, health insurance status and lipid-lowering medication use. * *P* < 0.05 after Bonferroni correction. **Supplementary Table 3.** Adjusted odds ratio in 5-year cognitive change per 10 mg/dL lipids at baseline across sensitivity analyses, OR (95%CI). Abbreviations: OR, odds ratio; CI, confidence interval; LDL-c, low-density lipoprotein cholesterol; HDL-c, high-density lipoprotein cholesterol. ^†^ Adjusted for baseline age, education, marital status, registered residence, body mass index, alcohol use, smoking status, diabetes, hypertension, social activity, health insurance status and lipid-lowering medication use. * P < 0.05, ** *P* < 0.01.

## Data Availability

The datasets that support the findings of the current study are available from the corresponding author on reasonable request. The data are publicly available and can be downloaded at http://charls.pku.edu.cn/index/zh-cn.html.

## Abbreviations

HDL-C: High-density lipoprotein cholesterol
LDL-C: Low-density lipoprotein cholesterol
TC: Total cholesterol
TG: Triglycerides
MST: Mental Status Test
WRT: Word Recall Test
