# Supplementary material for: Sex-specific associations between lipids and cognitive decline in the middle-aged and elderly: a cohort study of Chinese adults
Source: Alzheimers Res Ther. 2020 Dec 7;12:164. doi: 10.1186/s13195-020-00731-1 (PMC7722300; doi:10.1186/s13195-020-00731-1)
Supplement: Supplementary file 1 — Additional file 1: Supplementary Table 1. TC, LDL-c levels in different HDL-c grades. Supplementary Table 2. Adjusted odds ratio in 5-year cognitive change in different plasma lipid levels across education levels, OR (95%CI. Abbreviations: OR, odds ratio; CI, confidence interval; LDL-c, low-density lipoprotein cholesterol; HDL-c, high-density lipoprotein cholesterol. † Adjusted for baseline age, marital status, registered residence, body mass index, alcohol use, smoking status, diabetes, hypertension, social activity, health insurance status and lipid-lowering medication use. * P < 0.05 after Bonferroni correction. Supplementary Table 3. Adjusted odds ratio in 5-year cognitive change per 10 mg/dL lipids at baseline across sensitivity analyses, OR (95%CI). Abbreviations: OR, odds ratio; CI, confidence interval; LDL-c, low-density lipoprotein cholesterol; HDL-c, high-density lipoprotein cholesterol. † Adjusted for baseline age, education, marital status, registered residence, body mass index, alcohol use, smoking status, diabetes, hypertension, social activity, health insurance status and lipid-lowering medication use. * P < 0.05, ** P < 0.01. [file 13195_2020_731_MOESM1_ESM.docx]

**Supplementary Tables**

**Supplementary Table 1** TC, LDL-c levels in different HDL-c grades

| Items | HDL-c | | |
| --- | --- | --- | --- |
|  | <40, mg/dl | 40~60, mg/dl | >60, mg/dl |
| Female |  |  |  |
| N | 693 | 1790 | 749 |
| TC, mean | 186.46 | 197.76 | 205.82 |
| LDL-c, mean | 105.37 | 125.41 | 122.00 |
| Male |  |  |  |
| N | 947 | 1711 | 782 |
| TC, mean | 180.93 | 187.37 | 199.02 |
| LDL-c, mean | 107.05 | 116.64 | 112.39 |

**Supplementary Table 2** Adjusted odds ratio in 5-year cognitive change in different plasma lipid levels across education levels, OR (95%CI)

|  | Illiterate ^†^ | | | Primary school ^†^ | | | Middle school and above ^†^ | | |
| --- | --- | --- | --- | --- | --- | --- | --- | --- | --- |
|  | Male | Female |  | Male | Female |  | Male | Female |  |
| Global cognitive |  |  | |  |  | |  |  | |
| Total cholesterol | 1.04(0.96,1.12) | 1.04(1.00,1.08) | | 1.00(0.97,1.03) | 1.01(0.97,1.04) | | 0.99(0.97,1.03) | 1.04(0.99,1.09) | |
| <200 | ref | ref | | ref | ref | | ref | ref | |
| 200~239 | 1.21(0.64,2.28) | 1.17(0.85,1.61) | | 1.00(0.78,1.29) | 0.93(0.69,1.25) | | 0.96(0.73,1.26) | 1.02(0.69,1.51) | |
| 240+ | 1.04(0.37,2.98) | 1.34(0.87,2.05) | | 1.13(0.76,1.68) | 1.18(0.76,1.82) | | 1.03(0.70,1.50) | 1.28(0.79,2.07) | |
| LDL-c | 1.06(0.98,1.14) | 1.03(0.99,1.08) | | 1.00(0.97,1.03) | 1.01(0.97,1.04) | | 0.98(0.95,1.02) | 1.05(1.00,1.10) | |
| <100 | ref | ref | | ref | ref | | ref | ref | |
| 100~129 | 1.41(0.76,2.60) | 1.25(0.87,1.80) | | 1.00(0.77,1.31) | 1.34(0.97,1.85) | | 0.87(0.65,1.15) | 1.26(0.81,1.98) | |
| 130~159 | 1.25(0.57,2.74) | 1.34(0.89,2.01) | | 0.86(0.64,1.17) | 1.08(0.76,1.52) | | 0.88(0.61,1.15) | 1.61(0.99,2.59) | |
| 160~189 | 1.72(0.35,8.40) | 1.23(0.70,2.16) | | 1.10(0.68,1.78) | 1.21(0.72,2.03) | | 0.81(0.49,1.34) | 1.99(1.10,3.60) * | |
| 190+ | 1.66(0.35,7.83) | 2.13(1.02,4.46) * | | 0.98(0.52,1.84) | 1.17(0.60,2.29) | | 1.05(0.55,2.01) | 0.89(0.35,2.28) | |
| HDL-c | 0.85(0.71,1.02) | 1.10(0.98,1.24) | | 0.95(0.88,1.02) | 1.00(0.92,1.10) | | 0.98(0.91,1.06) | 1.11(0.99,1.25) | |
| <40 | ref | ref | | ref | ref | | ref | ref | |
| 40~59 | 0.91(0.48,1.76) | 1.28(0.87,1.88) | | 0.76(0.57,1.02) | 0.90(0.65,1.24) | | 0.84(0.64,1.11) | 1.73(1.08,2.76) * | |
| 60+ | 0.54(0.25,1.15) | 1.65(1.04,2.62) | | 0.72(0.52,1.00) | 1.01(0.69,1.47) | | 0.94(0.65,1.35) | 1.59(0.95,2.66) | |
| Triglycerides | 1.03(0.99,1.07) | 1.00(0.98,1.01) | | 1.00(0.99,1.02) | 1.00(0.99,1.02) | | 1.00(0.99,1.02) | 0.99(0.97,1.01) | |
| <150 | ref | ref | | ref | ref | | ref | ref | |
| 150~199 | 1.47(0.59,3.66) | 0.91(0.59,1.39) | | 1.09(0.77,1.53) | 1.13(0.76,1.69) | | 0.82(0.55,1.22) | 0.94(0.59,1.49) | |
| 200+ | 1.09(0.45,2.66) | 0.72(0.49,1.06) | | 1.15(0.80,1.64) | 0.94(0.66,1.33) | | 1.08(0.78,1.50) | 0.84(0.53,1.34) | |
| Mental status |  |  | |  |  | |  |  | |
| Total cholesterol | 1.03(0.97,1.10) | 1.03(0.99,1.08) | | 1.00(0.97,1.03) | 1.01(0.98,1.05) | | 0.99(0.96,1.02) | 1.06(1.00,1.11) | |
| <200 | ref | ref | | ref | ref | | ref | ref | |
| 200~239 | 0.96(0.51,1.84) | 1.15(0.85,1.56) | | 1.21(0.93,1.57) | 1.00(0.74,1.34) | | 1.15(0.88,1.51) | 1.58(1.07,2.34) | |
| 240+ | 0.94(0.44,1.99) | 1.28(0.83,1.97) | | 0.94(0.64,1.40) | 1.30(0.85,1.98) | | 0.85(0.56,1.29) | 1.35(0.82,2.24) | |
| LDL-c | 1.05(0.99,1.13) | 1.03(0.99,1.07) | | 1.00(0.96,1.03) | 1.01(0.98,1.05) | | 0.98(0.95,1.02) | 1.08(1.03,1.14) * | |
| <100 | ref | ref | | ref | ref | | ref | ref | |
| 100~129 | 1.65(0.89,3.07) | 1.15(0.81,1.63) | | 1.05(0.81,1.37) | 1.38(0.99,1.92) | | 0.86(0.65,1.14) | 1.37(0.88,2.12) | |
| 130~159 | 1.15(0.53,2.50) | 1.25(0.84,1.86) | | 0.93(0.68,1.28) | 1.11(0.78,1.58) | | 0.98(0.68,1.41) | 2.01(1.22,3.30) * | |
| 160~189 | 1.59(0.54,4.71) | 1.23(0.70,2.18) | | 0.97(0.59,1.59) | 1.35(0.80,2.27) | | 0.72(0.40,1.29) | 2.43(1.31,4.50) * | |
| 190+ | 2.40(0.81,7.08) | 1.53(0.81,2.90) | | 0.82(0.38,1.77) | 1.11(0.53,2.30) | | 0.72(0.35,1.51) | 1.04(0.37,2.95) | |
| HDL-c | 0.84(0.70,1.00) | 1.11(0.99,1.25) | | 0.99(0.92,1.06) | 0.99(0.91,1.09) | | 0.92(0.85,1.00) | 1.01(0.91,1.13) | |
| <40 | ref | ref | | ref | ref | | ref | ref | |
| 40~59 | 0.51(0.27,0.95) | 1.28(0.88,1.87) | | 0.82(0.62,1.09) | 0.81(0.59,1.13) | | 0.83(0.62,1.10) | 1.80(1.14,2.84) * | |
| 60+ | 0.38(0.19,0.79) * | 1.55(0.98,2.44) | | 0.84(0.61,1.17) | 1.03(0.71,1.49) | | 0.77(0.54,1.10) | 1.26(0.75,2.13) | |
| Triglycerides | 1.02(0.99,1.06) | 0.99(0.98,1.01) | | 1.00(0.98,1.01) | 1.01(0.99,1.02) | | 1.01(0.99,1.02) | 1.00(0.98,1.02) | |
| <150 | ref | ref | | ref | ref | | ref | ref | |
| 150~199 | 1.35(0.54,3.36) | 1.08(0.75,1.58) | | 1.08(0.74,1.58) | 1.55(1.05,2.29) | | 0.87(0.60,1.27) | 1.00(0.64,1.55) | |
| 200+ | 1.73(0.80,3.74) | 0.77(0.52,1.14) | | 1.05(0.73,1.50) | 1.15(0.81,1.62) | | 1.11(0.78,1.58) | 0.97(0.61,1.52) | |
| Episodic memory |  |  | |  |  | |  |  | |
| Total cholesterol | 1.01(0.93,1.09) | 1.01(0.97,1.04) | | 1.00(0.97,1.04) | 1.01(0.97,1.04) | | 1.00(0.96,1.03) | 1.00(0.95,1.05) | |
| <200 | ref | ref | | ref | ref | | ref | ref | |
| 200~239 | 1.27(0.66,2.45) | 1.02(0.74,1.40) | | 1.01(0.79,1.29) | 1.03(0.76,1.40) | | 1.01(0.77,1.32) | 0.76(0.50,1.14) | |
| 240+ | 1.03(0.35,3.05) | 1.08(0.74,1.58) | | 1.15(0.75,1.78) | 0.92(0.60,1.40) | | 0.95(0.64,1.43) | 1.19(0.71,1.99) | |
| LDL-c | 1.02(0.94,1.10) | 0.99(0.95,1.03) | | 1.01(0.98,1.05) | 1.01(0.97,1.05) | | 0.98(0.95,1.02) | 1.00(0.95,1.05) | |
| <100 | ref | ref | | ref | ref | | ref | ref | |
| 100~129 | 0.98(0.55,1.77) | 1.00(0.71,1.40) | | 0.96(0.73,1.26) | 1.46(1.06,2.01) | | 1.01(0.77,1.33) | 1.16(0.73,1.83) | |
| 130~159 | 1.38(0.62,3.03) | 0.99(0.66,1.50) | | 0.98(0.72,1.33) | 1.27(0.87,1.84) | | 0.85(0.59,1.23) | 0.99(0.61,1.62) | |
| 160~189 | 2.08(0.33,13.11) | 0.89(0.55,1.44) | | 1.13(0.67,1.90) | 1.27(0.71,2.26) | | 1.07(0.64,1.79) | 1.33(0.75,2.38) | |
| 190+ | 0.91(0.22,3.78) | 1.06(0.53,2.13) | | 1.79(0.92,3.46) | 0.91(0.41,2.03) | | 1.01(0.50,2.01) | 0.63(0.21,1.90) | |
| HDL-c | 0.85(0.73,0.99) * | 1.03(0.93,1.14) | | 0.96(0.89,1.03) | 1.00(0.91,1.09) | | 1.01(0.93,1.10) | 1.21(1.07,1.37) * | |
| <40 | ref | ref | | ref | ref | | ref | ref | |
| 40~59 | 1.04(0.56,1.92) | 0.92(0.64,1.32) | | 0.77(0.58,1.03) | 1.17(0.85,1.60) | | 0.90(0.69,1.19) | 1.36(0.83,2,24) | |
| 60+ | 0.50(0.23,1.08) | 1.18(0.79,1.76) | | 0.84(0.60,1.18) | 0.96(0.64,1.44) | | 1.07(0.75,1.52) | 1.93(1.11,3.37) * | |
| Triglycerides | 1.03(1.00,1.07) | 1.01(0.99,1.03) | | 1.00(0.98,1.02) | 1.00(0.98,1.01) | | 1.00(0.99,1.01) | 0.99(0.97,1.01) | |
| <150 | ref | ref | | ref | ref | | ref | ref | |
| 150~199 | 0.65(0.27,1.56) | 0.72(0.47,1.11) | | 0.87(0.60,1.24) | 0.83(0.58,1.19) | | 0.93(0.64,1.35) | 0.76(0.46,1.25) | |
| 200+ | 1.17(0.50,2.77) | 0.81(0.53,1.23) | | 1.04(0.71,1.53) | 0.80(0.55,1.17) | | 1.04(0.75,1.44) | 0.87(0.54,1.42) | |

Abbreviations: OR, odds ratio; CI, confidence interval; LDL-c, low-density lipoprotein cholesterol; HDL-c, high-density lipoprotein cholesterol.

^†^ Adjusted for baseline age, marital status, registered residence, body mass index, alcohol use, smoking status, diabetes, hypertension, social activity, health insurance status and lipid-lowering medication use.

* P<0.05 after Bonferroni correction.

**Supplementary Table 3** Adjusted odds ratio in 5-year cognitive change per 10 mg/dL lipids at baseline across sensitivity analyses, OR (95%CI)

|  | Global cognition^†^ | | | | | Mental status^†^ | | | | | Episodic memory^†^ | | | | |  |
| --- | --- | --- | --- | --- | --- | --- | --- | --- | --- | --- | --- | --- | --- | --- | --- | --- |
|  | Male | | Female | |  | Male | | Female | |  | Male | | Female | |  |  |
| **Sensitivity: Not adjusting the obesity** | | | | | | | | | | | | | | | |  |
| Total cholesterol | 1.00(0.98,1.02) | | 1.04(1.01,1.06) ** | | | 1.00(0.98,1.02) | | 1.04(1.01,1.07) ** | | | 1.00(0.98,1.02) | | 1.01(0.99,1.04) | | |  |
| LDL-c | 1.00(0.97,1.02) | | 1.04(1.01,1.06) ** | | | 1.00(0.98,1.02) | | 1.04(1.02,1.07) ** | | | 1.00(0.98,1.02) | | 1.01(0.98,1.03) | | |  |
| HDL-c | 0.96(0.92,1.01) | | 1.06(1.00,1.14) | | | 0.95(0.91,1.00) | | 1.06(1.00,1.13) | | | 0.98(0.93,1.03) | | 1.06(1.00,1.13) | | |  |
| Triglycerides | 1.00(0.99,1.01) | | 1.00(0.99,1.01) | | | 1.00(0.99,1.01) | | 1.00(0.99,1.01) | | | 1.00(0.99,1.01) | | 1.00(0.99,1.01) | | |  |
| **Sensitivity: Not adjusting the obesity and diabetes** | | | | | | | | | | | | | | | |  |
| Total cholesterol | 1.00(0.98,1.02) | | 1.04(1.01,1.06) ** | | | 1.00(0.98,1.02) | | 1.04(1.01,1.06) ** | | | 1.00(0.98,1.02) | | 1.02(0.99,1.04) | | |  |
| LDL-c | 1.00(0.97,1.02) | | 1.04(1.01,1.06) ** | | | 1.00(0.97,1.02) | | 1.04(1.02,1.07) ** | | | 1.00(0.98,1.03) | | 1.01(0.98,1.03) | | |  |
| HDL-c | 0.97(0.92,1.02) | | 1.07(1.00,1.14) | | | 0.95(0.91,1.00) | | 1.06(1.00,1.14) | | | 0.98(0.93,1.03) | | 1.06(1.00,1.13) | | |  |
| Triglycerides | 1.00(0.99,1.01) | | 1.00(0.99,1.01) | | | 1.00(0.99,1.01) | | 1.00(0.99,1.01) | | | 1.00(0.99,1.01) | | 1.00(0.99,1.01) | | |  |
| **Sensitivity: Not adjusting the obesity, diabetes and hypertension** | | | | | | | | | | | | | | | |  |
| Total cholesterol | 1.00(0.98,1.02) | | 1.04(1.01,1.06) ** | | | 1.00(0.98,1.02) | | 1.04(1.02,1.07) ** | | | 1.00(0.98,1.02) | | 1.02(0.99,1.04) | | |  |
| LDL-c | 1.00(0.97,1.02) | | 1.04(1.01,1.06) ** | | | 1.00(0.97,1.02) | | 1.04(1.02,1.07) ** | | | 1.00(0.98,1.02) | | 1.01(0.98,1.03) | | |  |
| HDL-c | 0.97(0.92,1.02) | | 1.06(1.00,1.14) | | | 0.95(0.91,1.00) | | 1.06(0.99,1.13) | | | 0.98(0.94,1.03) | | 1.06(0.99,1.13) | | |  |
| Triglycerides | 1.00(0.99,1.01) | | 1.00(0.99,1.01) | | | 1.00(0.99,1.01) | | 1.00(0.99,1.01) | | | 1.00(0.99,1.01) | | 1.00(0.99,1.01) | | |  |
| **Sensitivity: Not adjusting marital status** | | | |  | | |  | |  | | |  | |  | | |
| Total cholesterol | | 1.00(0.98,1.02) | | 1.03(1.00,1.05) * | | | 1.00(0.98,1.02) | | 1.03(1.01,1.06) ** | | | 1.00(0.98,1.02) | | 1.00(0.98,1.03) | | |
| LDL-c | | 1.00(0.97,1.02) | | 1.03(1.00,1.05) * | | | 1.00(0.97,1.02) | | 1.04(1.01,1.06) ** | | | 1.00(0.98,1.02) | | 1.00(0.97,1.02) | | |
| HDL-c | | 0.96(0.91,1.01) | | 1.06(1.00,1.13) | | | 0.95(0.90,1.00) | | 1.04(0.98,1.12) | | | 0.97(0.92,1.02) | | 1.06(1.00,1.13) | | |
| Triglycerides | | 1.01(0.99,1.02) | | 1.00(0.99,1.01) | | | 1.00(0.99,1.01) | | 1.00(0.99,1.01) | | | 1.00(0.99,1.01) | | 1.00(0.99,1.01) | | |
| **Sensitivity: Not adjusting health insurance status** | | | | | | |  | |  | | |  | |  | | |
| Total cholesterol | | 1.00(0.98,1.02) | | 1.03(1.00,1.05) * | | | 1.00(0.98,1.02) | | 1.03(1.01,1.05) * | | | 1.00(0.98,1.02) | | 1.00(0.98,1.03) | | |
| LDL-c | | 0.99(0.97,1.02) | | 1.03(1.00,1.05) * | | | 0.99(0.97,1.02) | | 1.03(1.01,1.06) ** | | | 1.00(0.98,1.02) | | 1.00(0.97,1.02) | | |
| HDL-c | | 0.96(0.91,1.01) | | 1.06(0.99,1.13) | | | 0.95(0.90,1.00) * | | 1.04(0.98,1.10) | | | 0.97(0.92,1.02) | | 1.06(1.00,1.13) | | |
| Triglycerides | | 1.01(0.99,1.02) | | 1.00(0.99,1.01) | | | 1.00(0.99,1.01) | | 1.00(0.99,1.01) | | | 1.00(0.99,1.01) | | 1.00(0.99,1.01) | | |
| **Sensitivity: Not adjusting marital and health insurance status** | | | | | | |  | |  | | |  | |  | | |
| Total cholesterol | | 1.00(0.98,1.02) | | 1.03(1.00,1.05) * | | | 1.00(0.98,1.02) | | 1.03(1.01,1.05) ** | | | 1.00(0.98,1.02) | | 1.00(0.98,1.03) | | |
| LDL-c | | 1.00(0.97,1.02) | | 1.03(1.00,1.05) * | | | 0.99(0.97,1.02) | | 1.03(1.01,1.06) ** | | | 1.00(0.98,1.02) | | 1.00(0.97,1.02) | | |
| HDL-c | | 0.96(0.91,1.01) | | 1.06(1.00,1.13) | | | 0.95(0.90,1.00) * | | 1.04(0.98,1.11) | | | 0.97(0.92,1.02) | | 1.06(1.00,1.13) | | |
| Triglycerides | | 1.00(0.99,1.02) | | 1.00(0.99,1.01) | | | 1.00(0.99,1.01) | | 1.00(0.99,1.01) | | | 1.00(0.99,1.01) | | 1.00(0.99,1.01) | | |
| **Sensitivity: Excluding participants with the obesity, diabetes or hypertension** | | | | | | | | | | | | | | | |  |
| Total cholesterol | 0.99(0.96,1.02) | | 1.05(1.01,1.08) ** | | | 1.00(0.97,1.02) | | 1.03(1.00,1.06) | | | 0.99(0.96,1.02) | | 1.03(1.00,1.06) | | |  |
| LDL-c | 0.98(0.95,1.02) | | 1.06(1.02,1.10) ** | | | 1.00(0.96,1.03) | | 1.04(1.01,1.08) * | | | 0.99(0.96,1.03) | | 1.03(0.99,1.07) | | |  |
| HDL-c | 0.95(0.89,1.01) | | 1.00(0.93,1.10) | | | 0.96(0.89,1.02) | | 1.03(0.95,1.12) | | | 0.95(0.89,1.02) | | 1.08(1.00,1.17) | | |  |
| Triglycerides | 1.01(0.99,1.02) | | 1.00(0.98,1.01) | | | 1.00(0.99,1.02) | | 0.99(0.97,1.01) | | | 1.00(0.98,1.01) | | 0.99(0.97,1.00) | | |  |

Abbreviations: OR, odds ratio; CI, confidence interval; LDL-c, low-density lipoprotein cholesterol; HDL-c, high-density lipoprotein cholesterol.

^†^ Adjusted for baseline age, education, marital status, registered residence, body mass index, alcohol use, smoking status, diabetes, hypertension, social activity, health insurance status and lipid-lowering medication use.

* P<0.05, ** P<0.01
